# Transfection in perfused microfluidic cell culture devices: A case study

**DOI:** 10.1016/j.procbio.2016.09.006

**Published:** 2017-08

**Authors:** William Raimes, Mathieu Rubi, Alexandre Super, Marco P.C. Marques, Farlan Veraitch, Nicolas Szita

**Affiliations:** Department of Biochemical Engineering, University College London, Bernard Katz Building, Gordon Street, London WC1H 0AH, United Kingdom

**Keywords:** Automated transfection, Microfluidic cell culture, Autologous cell therapy, Cell culture, Embryonic stem cells

## Abstract

Automated microfluidic devices are a promising route towards a point-of-care autologous cell therapy. The initial steps of induced pluripotent stem cell (iPSC) derivation involve transfection and long term cell culture. Integration of these steps would help reduce the cost and footprint of micro-scale devices with applications in cell reprogramming or gene correction. Current examples of transfection integration focus on maximising efficiency rather than viable long-term culture. Here we look for whole process compatibility by integrating automated transfection with a perfused microfluidic device designed for homogeneous culture conditions. The injection process was characterised using fluorescein to establish a LabVIEW-based routine for user-defined automation. Proof-of-concept is demonstrated by chemically transfecting a GFP plasmid into mouse embryonic stem cells (mESCs). Cells transfected in the device showed an improvement in efficiency (34%, n = 3) compared with standard protocols (17.2%, n = 3). This represents a first step towards microfluidic processing systems for cell reprogramming or gene therapy.

## Highlights

• Automated chemical transfection in a microfluidic device for long-term homogeneous cell culture.
• Transfection reagent mixture uniformly exposed to cells growing in the device.
• Embryonic stem cells transfected with GFP show improved efficiency and an increased median fluorescence intensity on-chip relative to 24 well plate.

## Graphical abstract

**Figure:**
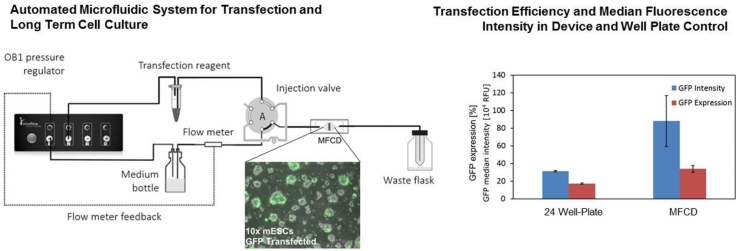

## 1 Introduction

There is a need to develop systems for the safe and economical production of cell therapies [1]. Autologous cell therapies only require a small starting cell population from a patient blood or skin sample. To derive iPSCs this somatic population needs to be transfected with pluripotent factors and maintained in stable long-term culture. Once derived, these cells can be further expanded and differentiated in downstream processing steps for transplantation back into the patient. First clinical trials for such autologous therapies are already underway in Japan for the treatment of macular degeneration. For this therapy, a small sheet consisting of retinal pigment epithelial (RPE) cells (5 × 10^4^ cells) is transplanted into the patient’s retina [2], [3]. A scale-down approach to bioprocessing particularly benefits treatments that require low cell input for transplant, such as required for an RPE-retina graft [4].

A key goal in bioprocessing is process integration to simplify unit operations, shorten residence times and reduce footprints [5], [6]. Integration can have additional advantages in cell processing, such as increasing cell viability by reducing the need for enzymatic detachment [7]. A recent example of integration in cell processing was demonstrated with cell expansion and differentiation in a single stirred reactor, where micro-carriers have been used to convert human pluripotent stem cells (hPSCs) into cardiomyocytes or neural progenitors. This was achieved with a high cell yield, low chance of contamination and a controlled aggregate size [6], [8]. A scale-down approach to autologous cell therapy presents an ideal platform to test and validate integration of unit operation steps. Transfection and long-term culture are important steps specific to iPSC therapies that could benefit from integration.

Electroporation is often regarded as having the highest efficiency of currently available micro-scale transfection approaches [9]. For example, cells were recently cultured on a porous polycarbonate substrate and transfected by localised electroporation to maintain high cell viability [10]. Integration of electrodes, however, typically increases the complexity of both device design and control [11]. Chemical transfection is a simpler method, and efficacy and viability continue to improve with each new commercial reagent [12]. It is important that transfecting agents are introduced in an automated fashion to minimise environmental fluctuations or operator bias, which are more likely to occur with manual procedures, and ultimately to improve robustness and reproducibility of the transfection process. A number of microfluidic culture devices have demonstrated chemical transfection of cultured cells [13], [14], [15], [16]. Examples include a digitally controlled cell-microchip with parallel circular culture chambers [13], and a self-contained system with near-chip peristaltic micro-pumps [16], both designed for combinatorial cell-based assays.

Integrated transfection devices described thus far compromise on two aspects essential for long-term stem cell culture: a uniform culture microenvironment and real-time analysis of growth kinetics and transfection outcome. To avoid compromise we integrate a two-position valve to automate the injection of a transfection reagent upstream of a microfluidic device that we previously developed and characterised for the long-term perfusion culture of adherent stem cells [17]. Our device offers uniform medium flow over the cell culture chamber and control over the dissolved gas concentrations [17], [18], and a fully automated and on-line culture monitoring system [19]. Furthermore, we successfully demonstrate transfection of mouse embryonic stem cells (mESCs), and we compare the efficiency of the transfection in the microfluidic device with a well-established manual culture protocol.

## 2 Materials and methods

### 2.1 Fabrication of the microfluidic cell culture device

The microfluidic culture device was fabricated according to Macown et al. [17]. Gaskets, gas-permeable lids and the microfluidic chip were cast from poly(dimethylsiloxane) (PDMS, Sylgard 184, Dow Corning, USA). A rigid polycarbonate holder in a screw-down aluminium frame was used to compress the microfluidic chip, which contained the fluidic channels and the culture chamber, against a tissue-culture polystyrene (TC-PS) microscope slide (260225, Elektron Technology Ltd, UK). The culture surface was 0.52 cm^2^ and the lid defined the height of the perfusion chamber at 450 μm, giving a chamber volume of ∼25 μL.

### 2.2 Pressure-Driven pumping system

**Fig. 1:**
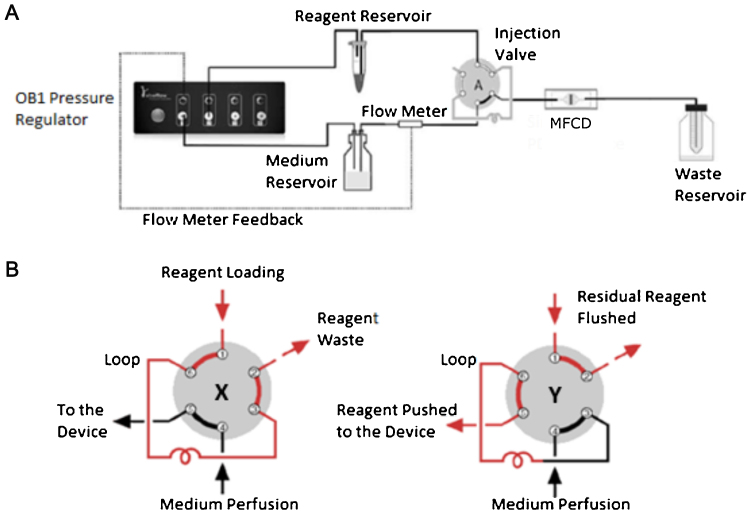
(A) A schematic of the perfusion system with feedback from the flow meter controlling the flow of culture medium into the microfluidic cell culture device (MFCD). Transfection mixture from the reagent reservoir is pushed into the injection loop by gas from the pressure regulator; once it has filled the loop, the valve will switch and reagent will move into the chip. (B) The two possible positions of the injection valve: X = reagent loading into loop, and Y = reagent injection into the microfluidic cell culture device.

The pressure-driven pumping system consisted of a gas supply (21% O_2_, 5% CO_2_, N_2_; BOC, UK) connected to a flow control system (OB1, Elveflow, France) which fed into a medium reservoir (DURAN^®^ bottle with GL-45 cap, Schott AG, Germany). The pressure was regulated by feedback control for a set flow rate of 5 μL min^−1^. The outlet of the medium reservoir was connected to a flow sensor (MFS 2, Elveflow, France), which fed into a low pressure, six-port injection valve (C22-3186EH, VICI AG International) with a 50 μL injection loop (Fig. 1A). The flow control system and injection valve were automated using LabVIEW (National Instruments, USA). The microfluidic culture device connected with the injection valve *via* a 10 cm long, 0.0635 mm inner diameter (ID) tubing (PEEK, IDEX Health & Science, USA). The device parts, medium reservoir and tubing were sterilised by autoclave and assembled in a biosafety cabinet under sterile conditions.

### 2.3 Cell culture

Mouse ESC were maintained as previously reported by Macown et al. [17]. The TC-PS slide was coated with 0.1% (w/v) gelatin (G1890, Sigma-Aldrich, UK) solubilised in Dulbecco’s Phosphate Buffer Solution (D1408, Sigma-Aldrich, UK) for 15 min at room temperature. The mESC culture medium consisted of knock-out Dulbecco’s modified Eagle medium (10829-018, Life Technologies, UK) supplemented with 15% v/v fetal bovine serum (26140-079, Life Technologies, UK). Priming and seeding of the microfluidic cell culture device was performed as described by Macown et al. [17]. Briefly, a suspension of mESCs (in culture medium) were seeded by pipette at a density of 2 × 10^5^ cells.cm^−2^. Cells were allowed to attach for 6 h in a 37 °C incubator before the start of perfusion. During perfusion the device was placed on the stage of an automated microscope (Eclipse Ti-E, Nikon Instruments, UK) at 37 °C in a cage incubator (Okolab, Italy).

### 2.4 Automated transfection in microfluidic culture device

Cells were transfected 24 h after the start of perfusion. The transfection mix consisted of a 2:3 ratio mixture of Lipofectamine^®^ 3000 (L3000-015, Life Technologies, UK) and GFP episomal plasmid (pCXLE-GFP, 27082, Addgene) in serum-free culture medium, following the protocol provided by Life Technologies (UK). 100 μL of the transfection mix, containing 1 μg plasmid DNA and 1.5 μL lipofectamine, was placed in a 1.5 mL Eppendorf^®^ microfluidic reservoir (KRXS, Elveflow, France). The reservoir was connected to the injection valve sample loop and to the pressure-driven pumping system (Fig. 1A). A LabVIEW routine (National Instruments, USA) automated the loading of the 50 μL injection loop from a second pressure channel, followed by a valve switch to flow the transfection mix to the culture chamber which held a maximum volume of ∼25 μL (Fig. 1B). Once the culture chamber was full of the transfection mixture, the pressure of the pumping system was switched off for 2 h. After this time culture medium perfusion was resumed at a flow rate of 5 μL min^−1^ to flush the transfection mix out of the culture chamber. Phase contrast microscopy images were taken 24 h post-transfection at 10×magnification using the microscope’s digital camera (DS-Fi1, Nikon Instruments, UK).

### 2.5 Transfection in well plates

Mouse ESCs were seeded in TC-PS 24-well plates (#3524, Corning, USA) at a density of 2 × 10^5^ cells.cm^−2^. The transfection mix described in section 2.4 (100 μL per well) was incubated for 10 min at room temperature and added to the culture wells containing 400 μL serum-free culture medium. Cells were then incubated for a 2 h period at 37 °C. After transfection, cells were incubated for a 24 h period in fresh medium containing serum. Pictures were taken using a digital fluorescence microscope (EVOS^®^ FL AMEFC4300, Life Technologies, UK).

### 2.6 Transfection analysis

Cells (both from device and well plates) were enzymatically detached (TrypLE select, 12563029, Life Technologies, UK) 24 h post transfection, and re-suspended in fresh medium before being analysed with a flow cytometer (BD-Accuri C6, BD Biosciences, UK). GFP expression was assessed with the FL1 detector of the flow cytometer. Transfected samples results were gated, excluding 99% of the un-transfected negative control population, to calculate the percentage of GFP-positive cells. All experiments were conducted in triplicates.

### 2.7 Injection time characterisation

A 20 μg mL^−1^ fluorescein solution (F6377-100G, Sigma-Aldrich) was used to measure injection times and concentrations in the culture chamber of the microfluidic device. Progression of the solution in the chamber was assessed by fluorescence microscopy imaging at 2× magnification. Images were acquired every minute during injection and wash-out of the solution, and every 30 min during the static period. Pixel intensities at different injection times were measured across the centre of the chamber using ImageJ [20]. A calibration curve was established measuring different concentrations of fluorescein in static conditions (no flow). To reduce the effect of photo-bleaching, the microscope epifluorescence lamp was only switched on during image acquisition.

### 2.8 Residence time distribution

A UV absorbance detector (D100 ActiPix, Paraytec Ltd, UK) for the detection of a tracer compound was connected to the effluent stream of the microfluidic cell culture device (Supporting Material A). Fluid flow was delivered by a low pressure neMESYS syringe pump (Cetoni GmbH, Germany). A 254 nm filter with a 12 nm bandwidth (254BP12, Omega Optical Inc., USA) was used to determine the step change between water and the tracer compound, a 200 mgL^−1^ aqueous solution of l-tryptophan (Sigma, UK). The tracer solution contained additionally 4 mM Allura red (Sigma, UK). A calibration curve was carried out by scanning different concentrations of the tracer. The cumulative response to the step change, F(t), was defined as:

(1) $$F\left(t\right)=\frac{C_{out}\left(t\right)}{C_{out,max}}$$

where C_out_(t) and C_out,max_ were the concentration of the tracer in the outlet at time, t, and the maximum tracer concentration, respectively. The step change function was plotted against the normalised residence time, defined as:

(2) $$\theta =\frac{Q}{V_{S}}.t$$

where Q and V_s_ were the flow rate and the setup volume, respectively. The setup volume comprised the volume of the microfluidic culture device, the downstream fluidic connectors and the measurement capillary (100 μm inner diameter, Postnova Analytics UK Ltd, UK).

The percentage of stagnancy (PS) was calculated as

(3) $$PS\left(\%\right)=\frac{\tau -\tau_{e}}{\tau }\times 100$$

where τ and τ_e_ were the residence time (V_s_/F) and the apparent mean residence time, calculated from the F(t), respectively. Measurements were performed in triplicates.

## 3 Results

### 3.1 Characterisation of residence times

**Fig. 2:**
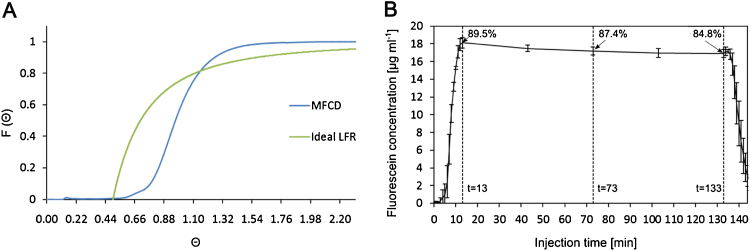
(A) Cumulative response, F(θ), of the normalised residence time, θ, of the microfluidic cell culture device (MFCD). The tracer concentration was 0.2 gL^−1^ delivered at a flow rate of 5 μL min^−1^. LFR − laminar flow reactor. (B) Residence time of fluorescein injected into a microfluidic cell culture device *via* an automated two-way valve at 5 μL min^−1^. After 13 min, maximal concentration (89.5%) was reached and flow was switched off. After 120 min flow resumed flushing the device. Images were taken every minute for injection/flushing and every 30 min during the static period. Values are the mean of three independent experiments, error bars display standard deviation.

In order to achieve proper characterisation of the transfection efficiency and to enable meaningful comparison with the batch operation modes of well plates, we conducted an analysis of the residence time distribution (RTD) in our setup (Fig. 2A). The RTD analysis of the culture device showed that the percentage of stagnancy was less than 1% (when compared with C_out,max_, as described in Eq. (1)). This indicates that in the culture chamber all cells are exposed to an equal concentration of transfection reagent. A fluorescein solution injection showed that 89.5% of the theoretical maximum concentration was reached ∼13 min post injection (Fig. 2B). Multiplying this time (∼13 min) with the set flow rate (5 μL min^−1^) gave us an accurate estimation of the pre-chamber volume (65 μL) for the LabVIEW-based automation of the setup. During the 2 h-long static phase, the relative fluorescein concentration decreased from 89.5% to 84.8% before the chamber was flushed (Fig. 2B).

### 3.2 Microfluidic cell culture device transfection

**Fig. 3:**
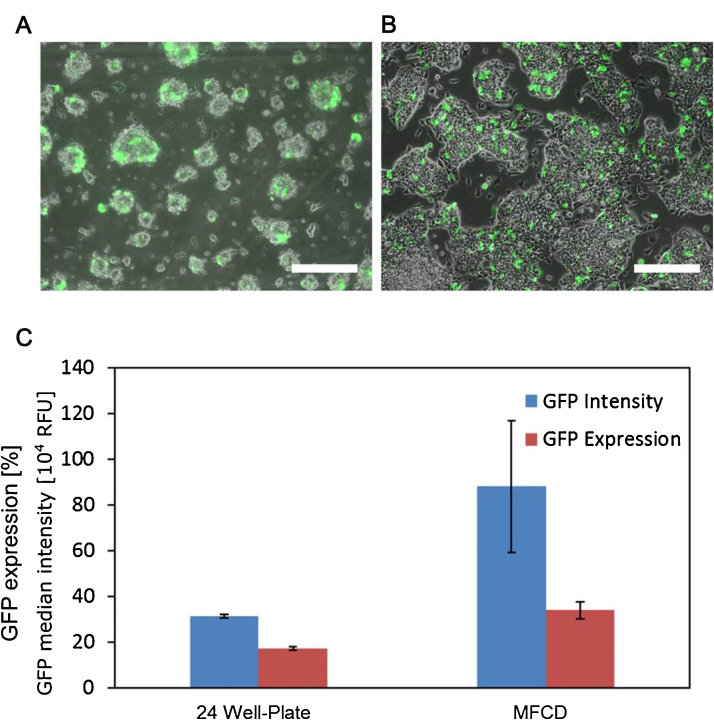
(A, B) 10 × FITC and phase contrast merged images of transfected cells in the microfluidic cell culture device (MFCD) and 24-well plate, respectively. Scale bar: 200 μm. (C) GFP expression and median fluorescence intensity for mESCs transfected with lipofectamine in the automated MFCD and a 24 well plate, measured using flow cytometry. Values are the mean of three independent experiments, error bars display standard deviation.

Twenty-four hours after transfection, GFP expression was visually assessed with fluorescence microscopy in the device (Fig. 3A) and the control wells (Fig. 3B). Additionally, flow cytometry was used to determine the percentage of GFP-positive cells in both cell populations (Supporting Material B). In the 24 well plate controls 17.2 ± 0.8% of the cells were GFP-positive. In the valve-mediated transfection 34.0 ± 3.9% of the cells in the device expressed GFP (Fig. 3C). A comparison of the average median fluorescence intensity (MFI) values of the GFP expressing populations indicates that cells transfected in the device had an MFI three times higher (8.81 × 10^5^ ± 2.89 × 10^5^ RFU) than those transfected in the well (3.13 × 10^5^ ± 0.7 × 10^4^ RFU) (Fig. 3C).

## 4 Discussion

A uniform concentration of transfection mix across the culture surface is necessary for efficient chemical transfection. In addition, a precise knowledge on the residence time of the transfection agent in the microfluidic culture chamber, i.e when the transfection agents enter and leave the culture chamber, is required to define the duration of reagent exposure. At the microscale, the flow in the culture device and tubing is laminar, therefore the delivery method must be precisely controlled. The parabolic flow profile of laminar flow systems ‘smears’ the distribution of reagent concentrations in flow. Calculating the time required for the transfection reagent to move from the injection valve to the culture chamber by dividing the ‘post-valve’ volume of the tubing by the flow rate during perfusion (5 μL min^−1^) alone would therefore not provide an accurate knowledge of when the reagent would reach the chamber. Furthermore, calculated dead-volumes are only approximations; experimental determination was required to gain exact knowledge. As such the pre-chamber volume, i.e. the volume between the valve and the culture chamber, was determined by multiplying the post valve perfusion time with the set flow rate. This volume was applied in a LabVIEW event loop with real-time feedback from the flow meter each second (Supporting Material C). Once this volume reaches zero (as determined by the fluorescence signal) the maximum chamber reagent concentration has been reached and the system automatically stops perfusion. Following this, a countdown starts before perfusion is restarted. It is important to incubate transfection reagent with DNA prior to transfection to ensure formation of lipo-complexes, however leaving this >15 min can cause a loss in transfection efficiency. Using the automated valve system, we can ensure that the time taken for the reagent mix to reach the chamber (i.e. incubation time) remains below this protocol-specified maximum.

It was not possible to completely saturate the culture chamber with fluorescein solution (89.5%) due to diffusion into the carrier medium during the injection process. The Stokes-Einstein equation states that the diffusion coefficient is proportional to the squared velocity of the diffusing particles, which is inversely related to the size of the particle. A lipo-complex (DNA:Lipid, ∼300 nm) is larger than a fluorescein molecule (∼0.45 nm) therefore has a lower diffusion rate. Thus the fluorescein concentration in the chamber will be lower than that expected for the larger lipocomplex. The further reduction in concentration seen over the full time course of the static transfection period is due to a small amount of fluorescein diffusion out of the culture chamber. To reduce diffusion into the carrying medium during the injection process, the injection loop (50 μL) provided double the volume of reagent required to fill the chamber. This means that once the maximum concentration is reached and the incubation phase begins, this excess transfection reagent remains in the pre-chamber tubing. This will pass over the cells at the end of the static period and therefore may contribute to increased efficiency present in the culture chamber, though exposure time is minimal.

The 1.8-fold transfection efficiency increase found in the device compared to the well plate can be explained by both the difference in surface-area-to-volume ratio (SA:V) and the necessary dilution of transfection mix in the well plate. The microfluidic cell culture device present a SA:V five times greater than a traditional culture well in a 24 well plate with a working volume of 500 μL. This shortens dramatically the diffusion timescale of the DNA:lipid complexes to reach the cells. Shallow enclosed microfluidic channels provide a clear advantage over larger open culture wells, with working volumes kept to a minimal since evaporation is no longer a concern. An improved efficiency is obtained for a reduced transfection reagent input. This proof-of-concept thus successfully demonstrates the injection valve as a functional approach for automated *in-situ* transfection in the perfused microfluidic device.

We can estimate the diffusion coefficient of lipoplexes in medium (D) to be 10^−12^ m^2^ s^−1^ using the Stokes Einstein equation:

$$D=\frac{kBT}{6\pi pa}$$

Where T is the absolute temperature (310.15 K), kB is the Boltzmann constant, *p* the medium viscosity (assuming that of water, 691.6 10^−6^ Pa.s, at 37 °C) and a the particle radius (assuming spherical, 300 nm). We use D to compare the characteristic time (t) of diffusion in a two-dimensional plane between the medium height (H) in the chamber (∼450 μm) relative to the well plate (∼10 mm) using Einstein’s relation:

$$t=\frac{H^{2}}{2D}$$

This comparison shows that it takes over 400 times longer for lipoplexes to diffuse across the well relative to the culture chamber considering the difference in medium height alone. This can also explain the MFI difference between the two culture vessels; cells in the microfluidic culture device received a higher individual eGFP load and therefore expressed more GFP than cells in the well. Further investigation of diffusional properties may be necessary depending on the non-integrating reprogramming vector used (i.e. episomal, mRNA or viral) [21].

Luni et al. recently published the first example of iPSC conversion in a microfluidic device [22]. Micro-volume confinement resulted in increased efficiency of iPSC conversion over traditional well-plate methods due to an increased concentration of endogenous signalling molecules. The authors used an on/off pneumatic valve system for daily medium replenishment and transfection of reprogramming factors. In contrast, we present a more complex culture system, which caters for switching between static transfection and controlled medium perfusion. As a proof-of-concept we focus here on demonstrating the benefits to transfection efficiency at the micro-scale. As of yet, there has been no report of perfusion used for cell reprogramming. There are however a number of advantages to the use of microfluidic perfusion culture. For example, the expansion rate of iPSCs increased with continuous perfusion relative to periodic medium changes [23]. By disrupting endogenous signalling, perfusion can also allow for a clearer identification of reprogramming-enhancing extrinsic factors [24], [25].

## 5 Conclusions

We investigated transfection in a microfluidic device designed for long-term adherent cell culture as a first step towards realising a scale-down tool that mimics the process integration necessary to derive iPSCs from patient cell samples. A bespoke LabVIEW routine automates the delivery of small volumes of transfection reagents to a perfusion microfluidic device *via* a six-port injection valve. This system is capable to be customized to account for different flow rates or incubation times in order to personalise the transfection process. Applicability has been shown by chemically transfecting mouse embryonic stem cells with a GFP plasmid. A higher efficacy was obtained in our device (34%) compared to standard culture protocol (17.2%). The better performance of an automated setup, together with the advantages of microfluidic devices, will ultimately allow for these systems to be used for cell reprogramming or for gene correction therapies.

## Conflict of interest

A patent application has been filed by UCL Business, a wholly-owned subsidiary of UCL (http://www.uclb.com). The application number is PCT/GB2009/002778. The author Nicolas Szita may become potential beneficiary of that patent application in the future. There are no further products in development or marketed products to declare.
