# Supplementary material for: Transfection in perfused microfluidic cell culture devices: A case study
Source: Process Biochem. 2017 Aug;59(Pt B):297–302. doi: 10.1016/j.procbio.2016.09.006 (PMC5615110; doi:10.1016/j.procbio.2016.09.006)
Supplement: Supplementary file 1 [file mmc1.docx]

**Supporting Material** – Raimes et al., Transfection In Perfused Microfluidic Cell Culture Devices: A Case Study


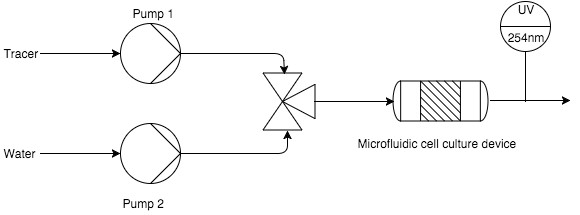


**Supporting Material A**. Experimental setup used for residence time distribution measurements. A three-way valve switches syringe pump controlled inlets from water to the tracer (L-tryptophan). A UV detector (Actipix™, Paraytec Ltd) situated at the outlet of the culture device measures the resulting step change function in the effluent as the tracer material flows through the device.


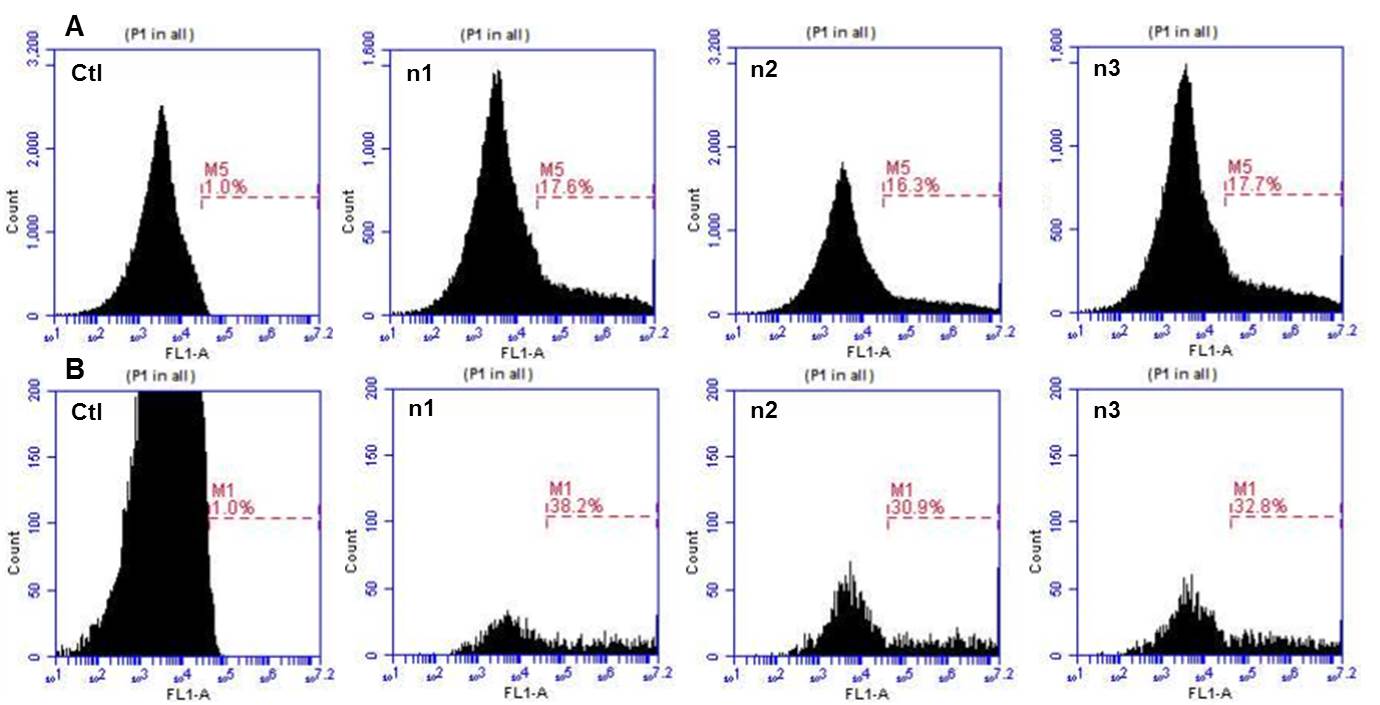


**Supporting Material B.** Raw histogram data from the flow cytometry analysis showing un-transfected cells (Ctl) and three separate runs in which mESCs were transfected with eGFP and Lipofectamine 3000 (n1, n2, n3). The x-axis (FL1-A) shows the relative GFP expression intensity of the cell population. **A**: 24-well plate. **B**: microfluidic cell culture device.


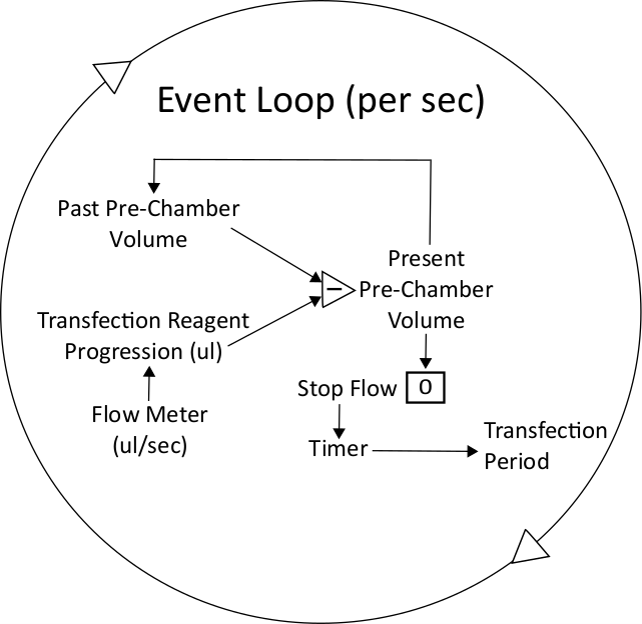


**Supporting Material C.** Flow chart of the LabVIEW control routine for automated transfection. Using real-time flow rate measurements and calculated fluidic volume, the progression of the injected volume is monitored in real-time. Flow is automatically stopped when the injection volume reaches the culture chamber and held static for a user-defined period.
